# Radiation dose and image quality comparison during spine surgery with two different, intraoperative 3D imaging navigation systems

**DOI:** 10.1002/acm2.12534

**Published:** 2019-01-24

**Authors:** Rami Nachabe, Keith Strauss, Beth Schueler, Mohamad Bydon

**Affiliations:** ^1^ Image Guided Therapy Systems Philips Healthcare Best The Netherlands; ^2^ Department of Radiology Cincinnati Children's Hospital Cincinnati OH USA; ^3^ Department of Neurologic Radiology Mayo Clinic Rochester MN USA; ^4^ Department of Neurologic Surgery Mayo Clinic Rochester MN USA

**Keywords:** cone‐beam CT, image quality, intraoperative 3D imaging, radiation dose, spine surgery

## Abstract

Careful protocol selection is required during intraoperative three‐dimensional (3D) imaging for spine surgery to manage patient radiation dose and achieve clinical image quality. Radiation dose and image quality of a Medtronic O‐arm commonly used during spine surgery, and a Philips hybrid operating room equipped with XperCT C‐arm 3D cone‐beam CT (hCBCT) are compared. The mobile O‐arm (mCBCT) offers three different radiation dose settings (low, standard, and high), for four different patient sizes (small, medium, large, and extra large). The patient's radiation dose rate is constant during the entire 3D scan. In contrast, C‐CBCT spine imaging uses three different field of views (27, 37, and 48 cm) using automatic exposure control (AEC) that modulates the patient's radiation dose rate during the 3D scan based on changing patient thickness. hCBCT uses additional x‐ray beam filtration. Small, medium, and large trunk phantoms designed to mimic spine and soft tissue were imaged to assess radiation dose and image quality of the two systems. The estimated measured “patient” dose for the small, medium, and large phantoms imaged by the mCBCT considering all the dose settings ranged from 9.4–27.6 mGy, 8.9–33.3 mGy, and 13.8–40.6 mGy, respectively. The “patient” dose values for the same phantoms imaged with hCBCT were 2.8–4.6 mGy, 5.7–10.0 mGy, and 11.0–15.2 mGy. The CNR for the small, medium, and large phantoms was 2.9 to 3.7, 2.0 to 3.0, and 2.5 to 2.6 times higher with the hCBCT system, respectively. Hounsfield unit accuracy, noise, and uniformity of hCBCT exceeded the performance of the mCBCT; spatial resolution was comparable. Added x‐ray beam filtration and AEC capability achieved clinical image quality for intraoperative spine surgery at reduced radiation dose to the patient in comparison to a reference O‐arm system without these capabilities.

## 1 INTRODUCTION

Pedicle screw malposition can lead to various complications such as vascular and visceral structure damage, or dural lesions and radiculopathy, which might require revision surgery.^1^ Image‐guided spine surgery using intraoperative three‐dimensional (3D)‐based navigation increases clinical accuracy of pedicle screw placement compared to free‐hand or fluoroscopy‐guided placement. Consequently, this improves patient safety.^2^, ^3^, ^4^, ^5^ In order to perform a spine surgery with navigation, an intraoperative 3D scan of the spinal region of interest is performed to use during navigation for localization of the instruments in the various 3D planes. At the end of the procedure, a second intraoperative 3D scan can be performed to assess the correct placement of introduced hardware as an alternative to radiographs or postoperative CT. Regardless of which type of camera navigation is used, it was demonstrated that the image quality of the intraoperative 3D scan can influence the accuracy of navigation.^6^, ^7^

Proper management of patient radiation dose is necessary. While use of 3D imaging‐based navigation decreased periprocedural radiation dose to staff, patient radiation dose increased.^8^ Better configuration of imaging equipment may achieve ALARA (as low as reasonably achievable) patient radiation doses.^9^ This is particularly important for the pediatric population with a higher risk of radiation‐induced cancer.^10^, ^11^

A mobile O‐arm system is a commonly used intraoperative 3D imaging system during spine surgery.^12^, ^13^ Other intraoperative 3D imaging systems have been widely used such as the Iso‐C 3D and the AiroCT.^12^, ^13^ Intraoperative 3D imaging improves the accuracy compared to preoperative 3D registration as well as the time per screw placement, which significantly reduces radiation dose to the patients.^14^ Other studies have investigated the patient's radiation dose from available systems and focused on the care needed to insure patient safety.^15^, ^16^ While some radiation dose reduction during intraoperative 3D navigation compared to C‐arm fluoroscopy‐guided spine surgery may occur,^17^ complex spine deformity remains a surgery where radiation dose to the patient is still significant when performed with intraoperative 3D navigation compared to other conventional techniques.^18^, ^19^

A hybrid operating room (OR) is a surgical suite containing an integrated C‐arm system within the room construction. Hybrid ORs are used in vascular surgery and have shown radiation dose reduction to the patient^20^ The benefit of using imaging equipment within a hybrid OR for pedicle screw placement and assessment with intraoperative 3D imaging has been documented.^21^, ^22^ Such benefits include the seamless integration of the C‐arm and the surgical table within the OR suite, the robotic movement of the C‐arm, and the ability to intraoperatively replace/adjust misplaced screws that could potentially require revision surgery after discovery during follow‐up CT imaging after closure. However, these studies have not investigated the corresponding radiation dose.

This study compares the management of radiation dose and simple analysis (measurements readily made in the field) of image quality of the planning phase of 3D scanning for navigation planning with a hybrid OR robotic ceiling‐mounted 3D cone‐beam CT unit (hCBCT)^21^ to that of a mobile O‐arm unit (mCBCT) as a reference.

## 2 MATERIALS AND METHODS

### 2.A Imaging devices

**Figure 1:**
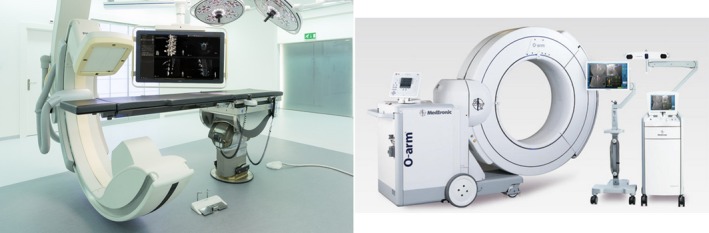
Hybrid operating room with a surgical table and a ceiling‐mounted motorized C‐arm Philips AlluraClarity system with augmented reality navigation camera mounted in the C‐arm detector and displayed on the hanging medical monitor (on the left). Medtronic O‐arm O2 system with infrared navigation camera system and medical imaging display carts (on the right). Courtesy of Philips Healthcare and Medtronic.

The different design technologies found in an O‐arm O2 (Medtronic, Littleton, MA) and an XperCT Augmented Reality Surgical Navigation System (Philips Healthcare, Best, the Netherlands) are compared (Fig. 1). Both systems use a flat panel detector, which enables two‐dimensional fluoroscopy as well as 3D CBCT. The mCBCT system is mounted on casters while the hCBCT unit is a robotized ceiling‐mounted C‐arm system. The source to detector distance (SID) during 3D imaging is fixed at 119 cm on both systems. Both systems’ hardware specifications are detailed in Table 1. The large focal spot was selected, 0.7 and 1.2 mm, respectively on the hCBCT and mCBCT during 3D CBCT imaging.

**Table 1:** 3D cone‐beam CT scanning parameters and reconstructed volumes of the various tested spine protocols on Philips hCBCT and O‐arm mCBCT for spine intraoperative imaging

	hCBCT 27 cm	hCBCT 37 cm	hCBCT 48 cm	mCBCT LD	mCBCT SD	mCBCT HD
Gantry size (cm)	85			70		
x‐ray focal spots size (mm)	0.4 and 0.7			0.6 and 1.2		
Detector size (cm)	40 × 30			40 × 30		
Detector pixel matrix	2480 × 1920			2038 × 1536		
Pixel size (mm)	0.154			0.194		
Detector 3D digital data conversion (bits)	16			12		
3D scan rotation (degrees)	180			360		
Number of projections	482	302	302	391	391	750
3D scan time (sec)	8	10	10	13	13	25
Cu beam filtration (mm)	0.4			0.1		
Antiscatter grid ratio	15:1			12:1		
kV	120			120		
mAs	Variable based on estimated patient thickness by the AEC. Ranges within 50–325			64/80/160/200 for S/M/L/XL thickness	128/200/320/400 for S/M/L/XL thickness	188/300/480/600 for S/M/L/XL thickness
CBCT matrix size	256^3^	383^3^	512^2^ × 396	512^2^ × 198		
CBCT voxel size (mm^3^)	0.492^3^	0.452^3^	0.492^3^	0.415 × 0.415 × 0.833		
CBCT diameter‐length (cm)	12.6–12.6	17.3–17.3	25.2–19.5	21.2–16.5		

LD, low dose; SD, standard dose; HD, high dose; S, small; M, medium; L, large; XL, extra large; AEC, automatic exposure control; CBCT, cone‐beam computed tomography.

Both systems use several preprogrammed anatomical locations for example, head, chest, abdomen, etc., for 3D acquisitions. Since this study focuses on lumbar spine imaging, the “lower torso/hip” and “Spine” protocols were chosen for the mCBCT and hCBCT, respectively. Two different parameters are used for 3D acquisitions on the mCBCT: four patient thickness [small (S), medium (M), large (L), or extra large (XL)] and three manual dose settings adjusted for the previously indicated patient thickness [low dose (LD), standard dose (SD) and high dose (HD)]. hCBCT has three selectable modes corresponding to the imaging field of view (FoV) of 27, 37, and 48 cm diagonal dimension. Automatic exposure control (AEC) capability, present on the hCBCT unit eliminates the need for the operator to select a patient thickness.

The AEC modulates the x‐ray tube current, which varies the dose rate to the patient for each angle of rotation. This maintains a relatively uniform detector dose rate despite varying patient thickness due to the rotation of the x‐ray beam about the patent. Units without AEC capability, require the operator to manually select a constant dose rate prior to a scan resulting in a relative constant dose rate to the patient and varying dose rate to the detector due to the elliptically shaped patient. The chosen dose level by the operator either improves image quality by reducing noise (more dose), or reduces image quality (less dose).^23^, ^24^, ^25^ Table 1 summarizes the difference in acquisition protocols and system specifications between the mCBCT and hCBCT units.^26^, ^27^ Note that for other applications than spine (e.g., pelvis) imaging, the mCBCT offers the largest CBCT diameter reconstruction of 40 cm.

This study was conducted in a hybrid OR room equipped with a surgical table (Alphamaquet 1150, Maquet AG, Switzerland) connected to a motorized ceiling‐mounted C‐arm flat detector system hCBCT. The mCBCT system was wheeled into the hybrid OR room to allow imaging with the same OR table.

### 2.B Phantoms

**Figure 2:**
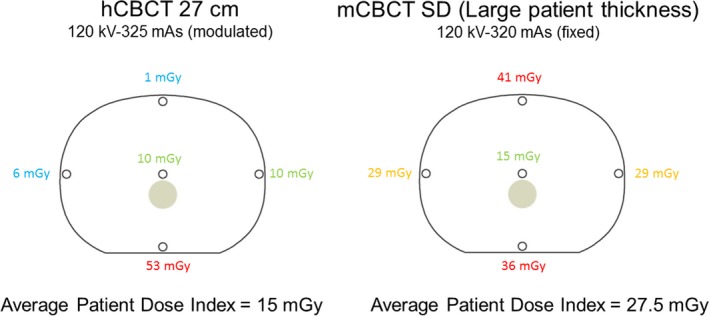
Example of measured dose value on the largest CIRS phantom size at each pin using hCBCT 27 cm FoV (left) and mCBCT SD for L patient thickness (right). The average patient dose index of each cross‐sectional area is listed below the phantom illustrations.

Radiation dose estimations were measured in three CIRS abdominal tissue equivalent CT dose phantoms, models 007TE‐03, ‐05, and ‐07 (CIRS, Norfolk, VA), which contain a rod designed to mimic the attenuation of the spine within the abdomen. Five holes, one at the center, and a top, bottom, left, and right‐side hole 1 cm below the surface of the phantom, allow insertion of a dosimeter probe (Fig. 2). The three phantoms have anteroposterior and lateral dimensions of 25.5 × 32.5, 18.5 × 24, and 14.0 × 18.0 cm, which represent an average adult, young teenager, and young child patient, respectively.^28^ Each phantom is 15 cm in length. Additionally, a Nuclear Associates 76‐415 body phantom (32 cm diameter cylinder with 15 cm length) was used to record dose exposures with an active length 100 mm 20X6‐3CT pencil ion chamber probe placed at each of the phantom 5 holes. This phantom is designed to model the adult trunk.

**Figure 3:**
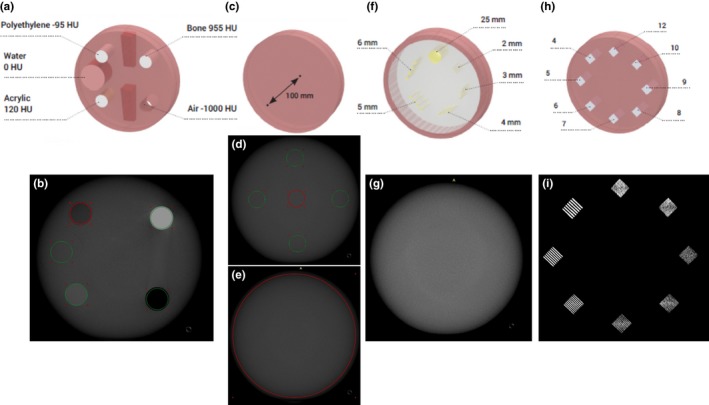
Sketch of the four modules in the ACR phantom to evaluate Hounsfield unit accuracy, noise and uniformity, contrast, and spatial resolution (from left to right top row) with corresponding CBCT imaging (bottom row).

Image quality assessment was completed with a CT ACR model 464 accreditation phantom, (Gammex, Middleton, WI) to measure CT Hounsfield unit (HU) accuracy, uniformity, and noise as well as low contrast and spatial resolution. Five cylinders with materials of known HU values (Fig. 3) are provided. Noise and uniformity are measured in a uniform HU cross‐sectional area of the phantom. Four cylindrical rods with five different diameter rods and a single larger rod all with a 6 HU contrast relative to the background material allow measurement of low contrast. Eight sets of line pairs (per cm) are used to measure spatial resolution. Figure 3 illustrates the spatial arrangement of these test objects, the object's size, and the corresponding images. Because of the small size of the ACR phantom, we used the small patient thickness (S) protocol on the mCBCT with a kV of 120 and mAs of 64, 128, and 188 for the LD, SD, and HD imaging protocol, respectively (Table 1). The kV on the hCBCT was also at 120 with a mAs set by the AEC as 85, 93, and 107 with the 48, 37, and 27 cm FoV imaging protocol, respectively. The cylindrical axis of the ACR phantom was iso‐centered when imaged with both systems. The boundary between Section 2 and 2.B of the ACR phantom was centered on the central ray of the cone beam.

### 2.C Radiation dose

The three CT dose phantoms were positioned on the table with the spine level centered within the clinical FoV of the resultant images. For each of the three phantoms, the corresponding patient thickness parameter was selected (i.e., S, M, and L) on the mCBCT system. Each phantom underwent dose measurements using all 3D acquisition protocols: LD, SD, and HD with the mCBCT and 27, 37, and 48 cm FoV with the hCBCT.

The dose within each of the five holes was measured, centered along the length of the 15 cm hole during a 3D scan using a black piranha and CT‐SD16 dosimeter probe with a disk sensor with an active volume of 10 mm^3^ (7 mm diameter and 0.25 mm thick), (RTI Electronics, Towaco, NJ). An average patient dose index estimate to the transverse cross‐section of the abdomen was calculated using the measured doses for the five measured point doses (1/3 of measured central dose plus 2/3 of the average of the four measured peripheral doses).^24^ Figure 2 depicts an example radiation dose measured at each location in the largest CIRS phantom using hCBCT 27 cm FoV and the SD setting on the mCBCT for large patient thickness.

Additionally, weighted computed tomography dose index (CTDI_w_) was calculated based on measurements on a 32 cm diameter cylinder 15 cm in length constructed of polymethyl methacrylate performed with a 20X6‐3CT pencil ion chamber with an active length of 100 mm.

### 2.D Contrast‐to‐noise ratio from the CIRS phantoms measurement

A high contrast‐to‐noise ratio (CNR) index was calculated: difference in mean HUs in a 7 cm^2^ circular region of interest (ROI) in the spine rod and “soft tissue” region below it within each CIRS phantom divided by the standard deviation of the soft tissue region. The relationship between CNR and weighted average measured dose provides an estimate on the compromise between image quality and radiation dose to the patient. For the sake of fair CNR comparison between both systems, the 3D datasets were formatted to the same slice thickness in the axial planes. The default image reconstruction algorithm for spine and bone imaging was used on both systems.

### 2.E Hounsfield unit accuracy form the ACR phantom

The HU accuracy was obtained by creating a ROI in each of the five rods of various materials available in the ACR phantom [cf. Fig. 3(a) and 3(b)]. The materials of known HU values are air (−1000 HU), polyethylene (−95 HU), water (0 HU), acrylic (120 HU), and bone (955 HU). A mean and standard deviation HU is documented for each ROI within each material for comparison with the actual HU.

### 2.F Uniformity, noise measurement, low contrast, and spatial resolution from the ACR phantom

Uniformity was evaluated by creating 5 ROI of 4 cm^2^ placed in the center, top, bottom, left, and right part of the ACR phantom [cf. Fig. 3(d)]. The uniformity was defined as the standard deviation of the HU within the five ROI divided by the HU difference between water and air.^29^

Noise was evaluated by creating a 105 cm^2^ ROI [cf. Fig. 3(e)] and was calculated as the standard deviation HU within the ROI divided by the HU difference between water and air.^29^

Low contrast is quantified by measuring the CNR of a 25 mm diameter pin compared to its background [cf. Figs. 3(f) and 3(g)]. Spatial resolution is based on the maximum resolvable detail of line pairs per cm (lp/cm) [cf. Figs. 3(h) and 3(i)].

### 2.G Statistical analysis

A weighted linear regression of the measured HU in the various materials with known HU in the ACR phantom was performed. The 95% confidence interval (CI) of the estimated slope and intercept were calculated as well as the adjusted‐R^2^ of the regression. A *P*‐value below 0.05 was considered for statistical significance. All calculations were performed by using a statistical software package (Matlab, version R2015a; Mathworks, Natick, MA).

## 3 RESULTS

### 3.A Radiation dose

**Figure 4:**
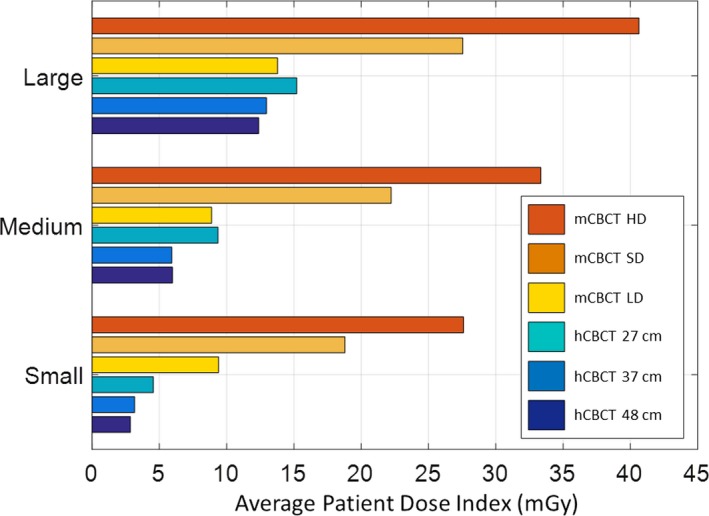
Patient dose index for each phantom size imaged with all hCBCT and mCBCT protocols.

The patient dose index range for the small, medium, and large CIRS phantoms imaged by the mCBCT for LD, SD, and HD was 9.4–27.6 mGy, 8.9–33.3 mGy, and 13.8–40.6 mGy, respectively. The patient dose index range for the same phantoms imaged by the hCBCT unit for 27, 37, and 48 cm FoV ranged from 2.8–4.6 mGy, 5.7–10.0 mGy, and 11.0–15.2 mGy, respectively. The patient dose index for the three phantoms from the hCBCT system was 30–17%, 64–30%, and 80–37% of the patient dose index of the mCBCT. Figure 4 illustrates these results.

**Figure 5:**
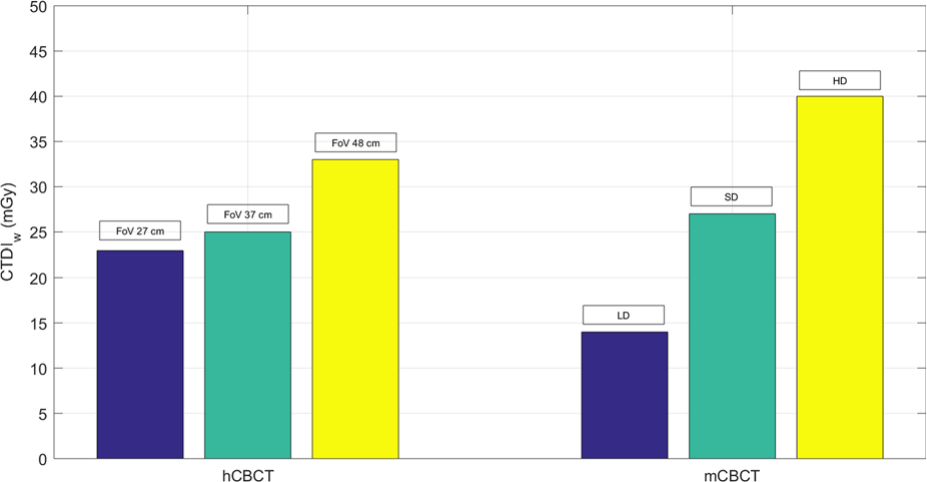
CTDI_w_ comparison of circular CTDI 32 cm body phantom from measurements performed with hCBCT and mCBCT protocols for large patient thickness.

The CTDI_w_ for the 27, 37, and 48 cm protocols on the hCBCT was 23, 25, and 33 mGy, respectively. The CTDI_w_ using the large patient protocols on the mCBCT was 14, 27, and 40 mGy, respectively (Fig. 5). Although the CTDI phantom size corresponds to a large patient similarly to the biggest CIRS phantom,^28^ we have made the measurements with the medium patient size protocol for comparison with available data in literature. The CTDI_w_ in a 32 cm circular CTDI phantom for the LD, SD, HD considering the medium patient protocols on the mCBCT was 7, 17, and 26 mGy, respectively.

### 3.B Contrast‐to‐noise ratio from the CIRS phantoms measurement

The CNR for the small, medium, and large CIRS phantoms imaged by the mCBCT for LD, SD, and HD was 9.7–16.7, 10.7–20.8, and 7.3–12.5, respectively. The CNR for the same phantoms imaged by the hCBCT unit for 27, 37, and 48 cm FoV was of the range 35.9–48.2, 32.2–41.2, and 18.7–31.8, respectively. The CNR for the small, medium, and large phantoms was 2.9–3.7, 2.0–3.0, and 2.5–2.6 times greater with the hCBCT compared to the mCBCT, respectively.

**Figure 6:**
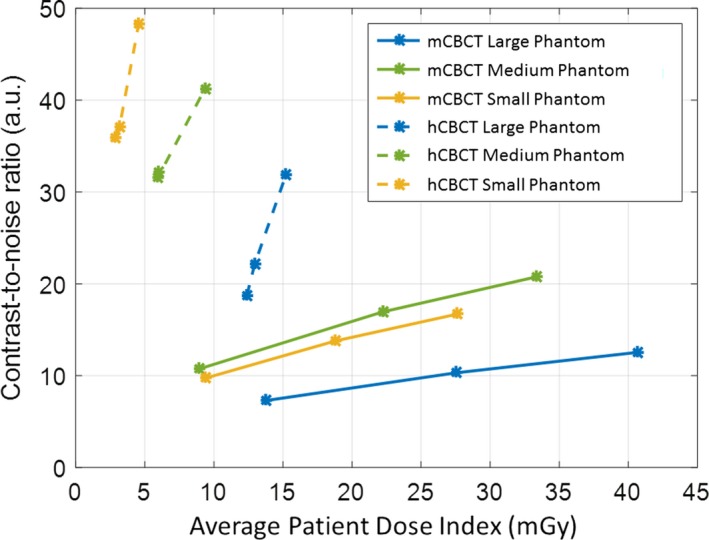
Contrast‐to‐noise ratio vs weighted average for each phantom size imaged with all hCBCT and mCBCT protocols.

Figure 6 displays the CNR vs patient dose index for all phantom sizes and imaging protocols. While CNR increases with increasing patient dose index for both imaging systems, the CNR of the mCBCT increases 0.4, 0.4, and 0.2 arbitrary units per mGy increase of patient dose index for the three phantom sizes, respectively. In comparison, the CNR of the hCBCT system increases 3.8, 2.1, and 4.6 arbitrary units per mGy increase in of patient dose index for the three phantom sizes, respectively.

### 3.C Hounsfield unit accuracy

**Figure 7:**
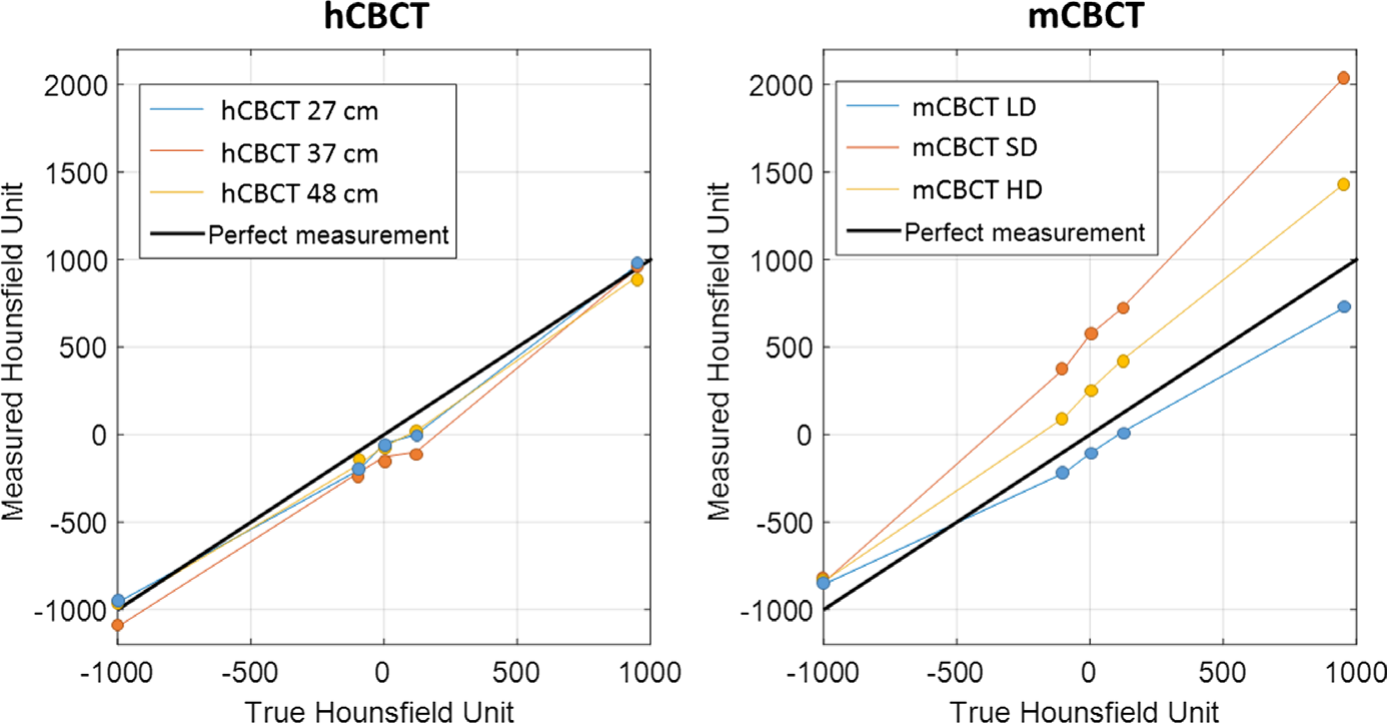
Hounsfield unit measurements compared to theoretical values. hCBCT on the left and mCBCT on the right. Each curve corresponds to an imaging protocol setting, except for the bold black line which corresponds to the theoretical values. For the mCBCT protocols, the higher the mAs of the imaging protocol, the higher the slope.

The HU accuracies of all CBCT performed on the ACR phantom are shown in Fig. 7 for all protocols on both systems as well as the desired measurement line with a slope of 1 and an intercept of 0.

The estimated slope of the mCBCT HU accuracy measurements was 1.16 (95% CI: [1.00 1.31]), 1.47 (95% CI: [1.32 1.62]), and 0.80 (95% CI: [0.67 0.93]) for the HD, SD, and LD protocols, respectively. The estimated intercept of the mCBCT HU accuracy measurements was 268 (95% CI: [199 337]), 566 (95% CI: [494 638]), and −98 (95% CI: [−153 −42]) for the HD, SD, and LD protocols, respectively. All estimated slopes and intercept parameters had a *P* < 0.05, and the adjusted R^2^ were 0.993, 0.996, and 0.990 for the HD, SD, and LD protocols on the mCBCT, respectively. The same measurements for hCBCT were: estimated slope 0.97 (95% CI: [0.80 1.12]), 1.04 (95% CI: [0.86 1.21]), and 0.96 (95% CI: [0.87 1.04]) for the 27, 37, and 48 cm protocols, respectively; estimated intercept −32 (95% CI: [−143 78]), −108 (95% CI: [−221 4]), and −57 (95% CI: [−112 −2]), for the 27, 37, and 48 cm protocols, respectively. While all estimated slopes again had a *P* < 0.05, the estimated intercept for the 48 cm barely showed statistical significance with a *P* = 0.046. The adjusted R^2^ were 0.989, 0.988, and 0.997 for the 27, 37, and 48 cm protocols, respectively.

### 3.D Uniformity, noise measurement, contrast, and spatial resolution from the ACR phantom

Uniformity, noise measurements, and spatial resolution are listed in Table 2. The uniformity and noise performance, 1.0–1.4% and 2.4–3.1% respectively on hCBCT, while 1.5–4.1% and 3.4–3.8% respectively on mCBCT. The resolvable high contrast detail for both systems is similar, namely ten line pairs per cm (Table 2). Since no low contrast rods were visible, the low contrast resolution is greater than 6 HU for both units. The CNR of the 25 mm diameter 6 HU pin for the hCBCT and mCBCT was 0.4–3.0 and 0.1–1.5 respectively as indicated in Table 2.

**Table 2:** Uniformity, noise values, spatial resolution, and CNR of the ACR 464 CT phantom for all protocols. The small patient thickness protocols of the mCBCT were selected given the 20 cm diameter of the imaged phantom

3D imaging protocol	Uniformity (%)	Noise (%)	Maximum resolvable detail (lp/cm)	CNR in 25 mm diameter rod
hCBCT 27 cm	1.4	3.1	9	5.1
hCBCT 37 cm	1.4	2.6	10	1.3
hCBCT 48 cm	1.0	2.4	9	0.8
mCBCT LD	4.1	3.8	10	0.1
mCBCT SD	2.1	3.4	10	0.4
mCBCT HD	1.5	3.4	10	1.5

LD, low dose; SD, standard dose; HD, high dose; lp, line pairs.

## 4 DISCUSSION

Two intraoperative imaging systems for spine surgery provided multiple imaging protocols and configurations yielding variable radiation dose to patients depending on the selected protocol. The patient dose index values (measured with CIRS phantoms) with the small, medium, and large phantoms from the hCBCT system were 30–17%, 64–30%, and 80–37% of the patient dose index values of the reference mCBCT. The CTDI_w_ comparison between both units needs to be compared for similar scanning length given the fact the measurement is performed with a pencil beam. Furthermore, given the size of the CBCT phantom, the large patient protocols on the mCBCT should be considered for comparison with the 37 cm FoV of hCBCT. The CTDI_w_ from the hCBCT for an adult size patient with adult technique was mGy which was thus 7.5% lower than the CTDI_w_ of the reference mCBCT. Few studies reported CTDI_w_ from the 32 cm CTDI phantom using the large patient size protocol with standard or high dose. It is important to note that because CTDIw is based on measurements with a pencil beam dosimeter, the radiation dose measured includes much more scatter compared to the single point measurement done on the CIRS phantoms making the comparison not suitable between both measurement techniques. Zhang et al. reported the lowest CTDI_w_ values of 6 mGy using the medium SD protocols; 5 and 10 mGy using the large patient with LD and SD protocols, respectively^.^ 26 Abul‐Kasim et al. reported a CTDI_w_ of 26 mGy using the large patient size SD protocol on the mCBCT.^30^ Petersen et al. reported a CTDI_w_ of 11 mGy using the medium patient size SD protocol on the mCBCT.^31^ Uneri et al. reported CTDI_w_ of 18 and 27 mGy when using the medium patient size with SD and HD protocols on the mCBCT.^32^ Our results are in agreement with all reported values in literature except those of Zhang, which reported lower values. The AEC from the hCBCT yields CTDI_w_ values, which are closest to the SD for large patient protocols on the mCBCT.

This study measured patient dose indices as a function of phantom size. Reported values in the mCBCT's radiation dose structured report (RDSR) were estimates of radiation dose delivered to a 32 cm diameter cylindrical phantom regardless of patient size (CTDI). The reported radiation dose output by the mCBCT system was for the large phantom and SD setting in Fig. 2 of 25.6 mGy, which was comparable to the measured patient dose index in this study of 25.5 mGy. However, when the small phantom was imaged to simulate a pediatric patient, the measured dose index, 18.8 mGy, exceeded the reported value of 11 mGy.

The RDSR reported dose index of the mCBCT under estimates pediatric patient doses for the same reason that the CT dose index reported by CT scanners, which assume a 32‐cm diameter cylindrical phantom, under estimates pediatric patient body doses during CT scanning.^33^ While the size‐specific dose estimate (SSDE) was developed to correct this systematic error for standard CT scanners in 2011, no such published conversion factors currently exist for the mCBCT. In comparison, the Philips C‐arm unit reports air Kerma at the interventional reference point of the fluoroscope and the Kerma‐area‐product in its RDSR. Since neither of these dose indices are defined for a rotational x‐ray beam, these two dose indices in the RDSR also failed to reasonably indicate patient dose for all patient sizes.

The 360‐degree rotation of the mCBCT yielded a radial dose distribution with least dose at the center of the body. The 180‐degree rotation of the hCBCT delivered an entrance dose from one lateral (LAT) to the opposite LAT projection through the posterior–anterior (PA) projection of the patient as illustrated in Fig. 2. This results in a nonuniform dose distribution similar to that of a single PA projection. The anterior surface of the patient does not receive an entrance dose, since the x‐ray tube does not rotate above the patient. Using the same dose rate delivers significantly less “average” radiation dose to the cross‐sectional plane illustrated in Fig. 2. This resulted in different organ doses between the two imaging systems depending on the cross‐sectional location of the organ. Centrally located organs received 50% more dose with the 360‐degree rotation. Organs peripherally located midplane (lateral positions) received approximately four times the dose than with the 360‐degree rotation. The anterior and posterior peripheral organ doses were 40 times greater and 66% for the 360‐degree rotation compared to the 180‐degree rotation.

In the example shown in Fig. 2, the patient dose for the hCBCT unit was approximately half of the mCBCT. Since the radiographic technique used by each unit was similar, 120 kV and 325 mAs for hCBCT while 120 kV and 320 mAs for mCBCT, and the source to image receptor distance is the same for each unit, the primary cause for the patient dose reduction is 0.4 vs 0.1 mm Cu filtration in the x‐ray beam. The additional filtration removes low energy x rays prior to the patient eliminating radiation dose delivered by x rays that would not reach the detector and contribute to image quality. This reduction of 50% was similar to a report of 40% reduction for similar thicknesses of Copper filtration.^34^ It is important to note that depending on the size of the FoV as well as the size of the phantoms and the degrees of rotation of the x‐ray tube that some of the surface locations of dose measurements in the phantoms might not be completely covered by entrance beam which results in reduced measured values.

The mCBCT used a fixed patient dose rate for a given patient based on the operator's choice of four patient thicknesses and three dose levels (Table 1). The PA and LAT projections were marginally over (increased dose) and under (reduced image quality) penetrated because of the same radiation exposure delivered by the mCBCT at each angle of the rotational scan. If the operator does not make these two choices correctly, the degree of error is increased, which can lead to compromised patient care. Clinical sites have developed patient weight ranges to assist the operator in classifying patient size.^35^

The AEC of the hCBCT modulated the patient dose rate in response to changes in patient thickness. Given the ellipsoid shape of the abdomen, the patient dose rate at the PA and LAT projections was at its lowest and highest values, respectively. The selected FoV determined the extent of the imaged anatomy (Fig. 2) beyond either side of the vertebral body, which was typically located in the center of the 3D scan. With smaller FoVs, the smaller cone beam reduced generation of scatter radiation, which improved image contrast. The limited fan beam rotation of hCBCT, that is, 180 degrees, resulted in more pronounced horizontal streak artifacts created by high‐attenuating materials such as the spine, which may in some instances obscure important anatomical details during clinical procedures.

A recent study by Vazquez et al. confirmed our findings with respect to HU accuracy for the mCBCT.^36^ The error of the measured HU increased as the attenuation in a given test pin increased. None of the mCBCT linear regressions had a slope of 1 with an intercept at 0 HU. In comparison, the measured linear regressions from the hCBCT unit had slopes and intercepts closer to 1 and 0, respectively.

The noise levels of the hCBCT images were reduced compared to the mCBCT images. While noise primarily decreased with higher radiation dose to the image receptor, careful choices of the reconstruction algorithm (unit's default spine/bone imaging algorithm) or reconstructed slice thickness (as defined in Table 1) also leveraged reductions in noise levels.

The measured uniformity of the mCBCT images was less than that of the hCBCT. The average energy of the x‐ray beam at greater depths in the patient increased due to attenuation of low energy x rays. This increased the value of HU at the periphery of large diameter objects.^37^ Clinically, these artifacts translate into over estimation of HU values of low‐attenuating structures (e.g., soft tissue) between high‐attenuating structures (e.g., bone). Beam‐hardening artifact correction algorithms are implemented as part of the reconstruction process only on the hCBCT to improve uniformity.

Low contrast resolution of 6 HU on the ACR phantom could not be visualized on either system. This corroborates with a reported failure of the mCBCT to visualize 10 HU.^37^ An increase in acquired projections can decrease noise and improve the visibility of low contrast soft tissues. For example, using 620 projections on the hCBCT unit for head imaging or a smaller FoV for spine imaging enabled a low contrast resolution of 3–5 HU.^21^

Spatial resolution on the mCBCT was 10 lp/cm, which was expected according to Nyquist sampling theory given a voxel dimension of 0.415 mm. The 27 and 48 cm FoVs of the hCBCT unit used a larger voxel size, 0.492 mm, resulting in spatial resolution of 9 lp/cm. A smaller voxel size might be required for the cervical spine where anatomical structures are smaller than in the thoracic or lumbar spine regions. For example, a voxel size as small as 0.130 mm would yield a spatial resolution ~20 lp/cm. However, this improved sharpness in the image increased noise (smaller voxels) at the expense of decreased contrast resolution.^38^

Intraoperative imaging of screw position is imperative to identify and move incorrectly placed screws without costly revision surgery.^39^ The diagnostic imaging performance of both units remains less than that of conventional CT.^40^ To date, intraoperative imaging with the mCBCT has not eliminated postoperative imaging with conventional CT.^8^ Further dose reduction is needed to make it attractive without compromising image quality, especially for cases requiring intervention at multiple levels of the spine like scoliosis. With dose reductions and improved image quality of technologies similar to those of hCBCT, clinical studies are needed to evaluate whether the patient radiation dose is low enough and imaging quality high enough to match the total periprocedural x‐ray radiation dose from x‐ray‐guided surgery or free‐hand with x ray.

Despite the fact that our phantom study had the advantage of experimental reproducibility at different dose settings and configurations, translation of the results to surgical settings is not straightforward. Quantitative metrics such as CNR, contrast, and spatial resolution are not easily correlated to the quality of an imaged patient spine. Hardware solutions using an angulated C‐arm to avoid x‐ray beam and metal implants collinearity^21^ as well as software for metal artifact reduction algorithms exist,^41^ but could not be evaluated due to absence of metal implants in available phantoms. In order to evaluate the image quality performance of CBCT for screw placement and the impact of metal artifact on diagnosing potential screw breach, the literature suggests performing receiver operating curve analysis where the screw placement evaluated in CBCT is compared to a gold standard such as CT.^22^ This analysis was beyond the scope of this study. Finally, this study compared the performance of only two different imaging units; a larger variety of imaging units are available. A dedicated phantom study with screws implanted in a spine model comparing a larger variety of imaging units is necessary to assess reduction of patient radiation doses while maintaining or improving image quality.

## 5 CONCLUSION

Two different imaging units, both capable of intraoperative 3D imaging with navigation during spine surgery were evaluated by comparing an estimate of dose to phantoms and associated image metrics produced by the two systems. Differences in the design of each system, mobile vs fixed installation, maximum kW, AEC, beam filtration, and image processing, and the manufacturer's guidance with respect to operational controls resulted in different measured patient radiation dose indices and image impressions achieved on the two systems.

## CONFLICT OF INTEREST

None of the authors were paid for this work.
